# Exercise Benefits Brain Function: The Monoamine Connection

**DOI:** 10.3390/brainsci3010039

**Published:** 2013-01-11

**Authors:** Tzu-Wei Lin, Yu-Min Kuo

**Affiliations:** 1 Institute of Basic Medical Sciences, National Cheng Kung University, Tainan 70101, Taiwan; E-Mail: s58991219@mail.ncku.edu.tw; 2 Department of Cell Biology and Anatomy, National Cheng Kung University, Tainan 70101, Taiwan

**Keywords:** exercise, brain function, monoamine

## Abstract

The beneficial effects of exercise on brain function have been demonstrated in animal models and in a growing number of clinical studies on humans. There are multiple mechanisms that account for the brain-enhancing effects of exercise, including neuroinflammation, vascularization, antioxidation, energy adaptation, and regulations on neurotrophic factors and neurotransmitters. Dopamine (DA), noradrenaline (NE), and serotonin (5-HT) are the three major monoamine neurotransmitters that are known to be modulated by exercise. This review focuses on how these three neurotransmitters contribute to exercise affecting brain function and how it can work against neurological disorders.

## 1. Introduction

Regular physical exercise has been proved to have therapeutic benefit [1], such as treating psychiatric illnesses [2,3,4,5,6,7], supporting brain injury recovery [8,9,10,11,12], and resisting neurodegenerative diseases [13,14,15,16,17,18]. The advantageous effects of exercise on brain functions have been attributed to increased capacities of metabolism reserve and antioxidation [19,20]. Furthermore, regulations of the secretion of neurotrophic factors, vasculotropic factors, inflammatory mediators, and neurotransmitters are also involved in exercise’s influence on brain function [21,22,23,24,25]. Among these effects, secretion of neurotransmitters, especially monoamines, have been linked to the exercise-induced neuronal adaptation. Interplay between exercise and monoamines was initially derived from the “Central Fatigue Hypothesis”, in which increased brain 5-HT release was found to be associated with central fatigue [26,27]. 5-HT is linked to fatigue because of its known effects on sleep, lethargy, and loss of motivation. The hypothesis prompted researchers to investigate the role of 5-HT in exercise-induced central fatigue [28,29,30,31,32,33]. Results suggest that exercise-induced central fatigue is more complex and depends on the intensity and duration of exercise. Overtraining exercise may stimulate hyperactivation of the monoamine systems which could cause fatigue [34,35,36]. Rodents that receive exhaustive treadmill exercise have higher 5-HT in the midbrain, striatum, hypothalamus and hippocampus [37]. The levels of dopamine are increased only in the striatum, although the levels of DOPAC, the major DA metabolite, are increased in both midbrain and striatum. On the other hand, exhaustive treadmill exercise reduces NE levels in the hypothalamus and brain stem [38]. Chronic moderate exercise is also known to stimulate the monoamine systems, but exercise of this sort does not induce central fatigue. Instead, chronic moderate exercise has been recognized as one of the most effective ways to enhance the adaptation/plasticity of the central nervous system (CNS). In addition, clinical studies suggest that monoamine systems play central roles resistance and recovery induced by chronic moderate exercise from various diseases like mental disorders [39] and Parkinson’s disease (PD) [40]. This article reviews recent studies that emphasize the effects of exercise on brain functions due to monoamine systems.

## 2. Physiological Properties of DA, NE and 5-HT

The catecholamines (DA, NE) and 5-hydroxytryptamine (5-HT) are the chief players of the monoamine neurotransmitter family. All three monoamines can be re-uptake by specific transporters from the synaptic spaces back into cytosol. Furthermore, they share common activation mechanism mediated through G protein-coupled receptors (GPCRs). We shall first briefly introduce the biosynthesis of DA, NE and 5-HT, as well as the biological properties of their receptors. 

### 2.1. Dopamine (DA) System

DA is synthesized from l-dihydroxyphenylalanine (l-DOPA), which is catalyzed from amino acid tyrosine by enzyme tyrosine hydroxylase. l-DOPA is then converted to DA by the catalysis of enzyme DOPA decarboxylase (or aromatic amino acid decarboxylase). In the CNS, dopaminergic neurons mainly reside in two nuclei of the midbrain: substantia nigra and ventral tegmental nucleus. Axons of the dopaminergic neurons in the substantia nigra project to the striatum (nigrostriatal pathway) which in turn is responsible for movement behavior. Axons of the ventral tegmental nucleus dopaminergic neurons project to the entire cortex (mesocortical pathway) and nucleus accumbens (mesolimbic pathway), which are involved in cognition and reward responses, respectively. 

There are five types of DA receptors (D1 to D5), which are simply categorized in two families of GPCR: D1-like and D2-like receptors. The D1-like receptor family contains D1 and D5 receptors that are coupled with G_s_ and G_q_ protein [41,42,43,44]. The D2-like receptor family comprises of D2, D3 and D4 receptors which couple with the G_i_ proteins to inhibit adenylyl cyclase [43,44]. D2-like receptors can be found on both pre- and post-synaptic terminals, while D1-like receptors are mainly located at post-synaptic sites [43]. The actions of DA receptors on membrane excitability are well defined in the striatal neurons [45,46,47,48,49]. Both D1 and D2 receptors are capable of modulating long-term depression; activation of D1 receptor enhances long-term depression, while activation of D2 receptor inhibits long-term depression [50]. Furthermore, D1 and D2 receptors modulate motor functions by modifying the excitatory transmission between the presynaptic cortical glutamatergic neurons and the post-synaptic striatal GABAergic neurons [46]. 

### 2.2. Norepinephrine (NE) System

NE is synthesized by further hydroxylation of DA. In the CNS, NE is released by the noradrenergic neurons which are mainly present in the locus coeruleus. The axons of noradrenergic neurons innervate the whole cerebral cortex, various subcortical areas, cerebellum and brain stem [51]. 

Traditionally, noradrenergic receptors are divided into three types: α_1_, α_2_ and β. So far, three α_1_ subtypes (α_1a_, α_1b_, α_1d_), four α_2_-receptor subtypes (α_2A–D_), and three β subtypes (β_1–3_) receptors have been identified; all of them are the members of the GPCR [52]. The α_1_ and β receptors are present primarily at the postsynaptic sites, whereas α_2_-receptors exist at both pre- and postsynaptic terminals. α_1_ receptor is the most abundant adrenergic receptor in the brain [53]. Binding of NE to α_1_ receptor, a G_q_ coupled receptor, activates phospholipase C, which in turn produces inositol 1,4,5-trisphosphate and diacylglycerol to regulate intracellular Ca^2+^ concentration and the activation of PKC. α_2_ Receptor, a G_i_ coupled receptor, whose main function is to increase glycogenesis in neurons [54]. β Receptor, linked to G_s_ protein, can either activate PKA through activating adenylyl cyclase and cAMP or cause apoptosis via the Src family tyrosine kinase-dependent pathway [55]. The activity of these receptors carries out a variety of tasks in the CNS.

### 2.3. Serotonin (5-HT) System

5-HT is synthesized from l-tryptophan, which is first catalyzed by tryptophan-5 hydroxylase to form 5-hydroxytryptophan and then further converted to 5-HT by aromatic l-amino acid decarboxylase. The axons of serotonergic neurons, primarily from the median and dorsal raphe nuclei, project to the entire CNS. 5-HT is regarded as the keystone in the psycho-emotional symptoms of depression and anxiety [56,57]; antagonists of 5-HT transporter have been widely used to ameliorate neuropsychiatric symptoms [58]. 

Seven classes of 5-HT receptors (5-HT_1_ to 5-HT_7_) have been identified. Except for the 5-HT_3_ receptor, which is a ligand-gated ion channel, all the other 5-HT receptors belong to GPCR family [59]. Previous efforts in clarifying the effect of exercise on brain function focus primarily on 5-HT_1A_, 5-HT_1B_ and 5-HT_2A_ receptors. Alterations of 5-HT_1A_, 5-HT_1B_ and 5-HT_2A_ levels have been linked to the development of anxiety, depression and psychiatric disorders such as schizophrenia [60,61,62,63,64,65,66,67,68,69,70], making these three types of receptor favorable targets for drug treatment. All six subtypes of 5-HT_1_ receptor (5-HT_1A–F_) are members of the G_i_-coupled receptor family and function to inhibit adenylyl cyclase activity. 5-HT_1A_ receptors are highly expressed not only at the somatodendritic sites (autoreceptor) of raphe nuclei, but also at the postsynaptic sites of limbic area, especially the hippocampus [59]. 5-HT_1B_ receptors function as the terminal autoreceptor and are enriched in the basal ganglia, striatum and frontal cortex. In addition, 5-HT_1B_ receptors may also behave as a terminal heteroreceptor to control the releases of acetylcholine, glutamate, DA, NE and GABA [59,71]. The 5-HT_2_ class consists of the 5-HT_2A_, 5-HT_2B_ and 5-HT_2C_ receptors. 5-HT_2A_ receptors are mainly located in the cortex, claustrum and basal ganglia. Activation of 5-HT_2A_receptors results inneuronal depolarization via the phospholipase C-Ca^2+^/PKC pathway [72]. 

## 3. Exercise Modulates the Activities of DA, NE and 5-HT Systems

A plethora of evidence from animal and human studies indicates that physical exercise improves health, both physically and mentally [73,74,75]. In the follow section, we will discuss how exercise, via the regulations of monoamine systems, enhances physical and mental adaptive capacities in order to cope with external/internal challenges and maintain homeostasis.

### 3.1. Exercise and DA System

Exercise is known to change the DA system in the CNS. Using a radioenzymatic assay followed by a thin-layer chromatography, the concentrations of DA are found to be upregulated in brain homogenates of rats subjected to eight weeks of food-reinforced running-wheel exercise, while a compensatory down-regulation of DA receptor densities is evident in these animals [76]. Upregulation of DA in the brain has been linked to exercise-induced higher levels of serum calcium, which is transported into the brain and affects calcium/calmodulin-dependent DA synthesis by activating the tyrosine hydroxylase enzyme [77]. Furthermore, the binding affinity between DA and DA receptor, determined by [3H]spiroperidol binding, is also increased by exercise training [78,79]. 

Interestingly, cerebral infarction induced by photothrombosis increases striatal DA content levels [80]. The infarction-induced increases of striatal DA level are reduced by eight days of running-wheel exercise initiated from two days after infarction. Furthermore, running-wheel exercise-induced increases of cortical levels of DA cannot be reproduced in the female R6/1 mice, a model of Huntington disease [81]. In a MPTP-induced PD animal model, treadmill exercise not only increases the stimulus-evoked DA release but also decreases the decay of DA in the dorsal striatum as determined by fast-scan cyclic voltammetry technique [82]. It has been shown that treadmill exercise counteracts the MPTP-induced motor dysfunction by increasing the levels of DA-D2R mRNA and decreasing the levels of striatal DA transporter protein [83]. More recently, upregulation of striatal dopamine D2 receptor by treadmill exercise in the MPTP-induced PD mouse model has been validated by positron emission tomography [84]. The beneficial effects of exercise, both wheel and treadmill running, can be confirmed in a different PD animal model—6-hydroxydopamine-induced dopaminergic neuron death [85,86,87]. In addition to the therapeutic effects, exercise also provides protection against neurotoxicity. Three months of wheel exercise prior to MPTP treatment protects dopaminergic neuron against MPTP-induced loss in the substantia nigra of mice [88]. Furthermore, in peripheral inflammation-induced PD animal model, four weeks of treadmill exercise before inflammation has been shown to prevent the inflammation-induced dopaminergic neuron loss [89]. 

Studies of patients with PD suggest that exercise may provide preventive and non-pharmaceutical therapeutic approach for PD. Six months of aerobics exercise significantly improves the executive movement of simple and complex motions in patients diagnosed with mild to moderate PD [90]. Additionally, in an epidemiological evaluation of physical activity in a cohort of more than 200,000 participants, exercise at moderate to vigorous levels is found to protect against PD [91]. Altogether, exercise not only modulates the direct action of DA system, but also protects DA neuron against toxic assaults.

### 3.2. Exercise and NE System

In response to external stimuli and the pressure of survival, animals adapt themselves physically and mentally. Exercise is known to enhance neuronal adaptation against harmful stimuli associated with stress. When subjected to inescapable tail shock, an elevation of c-Fos protein, an indication of neuronal activity, occurs in neurons of locus coeruleus [92]. Such stress-induced neuronal activation in the locus coeruleus is reduced by six weeks of wheel running, suggesting that exercise provokes neuronal adaptation in response to uncontrollable stress [92,93]. The protective mechanism of exercise against stress has been attributed to the expression of galanin in locus coeruleus [94]. Galanin hyperpolarizes noradrenergic neurons and inhibits locus coeruleus neuronal firing, leading to a suppression of NE release [95,96]. Reduced NE release from locus coeruleus to target areas, such as frontal cortex and amygdala, confines anxiety behavior [94]. Both chronic treadmill and running-wheel exercise increase the expression of galanin gene in the locus coeruleus [97,98,99,100]. Furthermore, the levels of plasma galanin are also increased in humans after acute exercise [101]. 

In addition to stress resistance, NE also participates in commanding the consolidation and retrieval of memory [102,103], especially emotional memory [104]. Loss of noradrenergic neurons in locus coeruleus has been implied to be involved in the symptoms of cognitive impairment in PD and Alzheimer’s disease [105,106]. Both chronic treadmill and wheel exercise increase the levels of NE in the pons-medulla and spinal cord as compared to sedentary controls [107]. Similarly, the levels of NE in brain regions that are linked to cognitive function, including hippocampus and central and medial amygdala, are elevated by chronic treadmill exercise [108]. Blockade of β-adrenoreceptors with various antagonists inhibits the chronic exercise-induced improvement of learning and memory in contextual fear conditioning and water maze tasks [100,109]. The memory performance in both amnestic mild cognitive impairment patients and control subjects is significantly enhanced if a single bout of aerobic exercise is given immediately after learning [110]. Meanwhile, the endogenous activity of NE is also increased by exercise, suggesting a potential linkage between NE and exercise-enhanced cognitive function.

### 3.3. Exercise and 5-HT System

The 5-HT system is modulated by exercise, though such modulation is brain-region-dependent and is affected by the intensity and duration of exercise. Four weeks of moderate treadmill exercise decreases the levels of 5-HT without affecting the metabolism of 5-HT in the hippocampus [111]. On the contrary, seven days of high-intensity treadmill exercise significantly increases the levels of hippocampal 5-HT [112]. Furthermore, four-week moderate treadmill exercise does not change 5-HT concentration in the amygdala [111]. The levels of 5-HT and its metabolite (5-HIAA) in cortex and striatum are significantly reduced in Huntington’s disease (R6/1) mice [81]. Four weeks of running-wheel exercise increases the striatal levels of 5-HIAA in the R6/1 female mice [81]. In the dorsal and median raphe nuclei, running-wheel exercise decreases the levels of 5-HT and 5-HT transporter mRNA, but increases the levels of 5-HT_1A_ receptor mRNA [113]. The concentrations of 5-HT in the brain stem and the hypothalamus are unaltered by wheel exercise [114]. These results indicate that the synthesis and secretion of 5-HT are affected by the intensity of exercise. The high levels of 5-HT induced by high intensity of exercise may also contribute to the central fatigue after endurance exercise. In an infraction animal model, eight days of running-wheel exercise, initiated from two days after infarction, increases the levels of 5-HT in the striatum [80]. Importantly, the levels of 5-HT positively correlate to the motor performance in rotarod test [80]. Moderate exercise has been suggested to serve as a treatment strategy for several psychiatric disorders. Although the mechanism is unclear, regulation of 5-HT receptors has been postulated as one of the potential mechanisms. Most previous studies examining the effect of exercise on 5-HT receptors only focused on 5-HT_1A_ and 5-HT_1B_ receptors, leaving the other subtype of 5-HT receptors largely unknown. The effect of exercise on 5-HT receptor is also brain-region-specific. For example, chronic wheel running is known to increase the levels of 5HT_1A_ receptor mRNA in the dorsal raphe nucleus [113], while the levels of such mRNA are decreased in the hippocampus [115]. Maternal separation-induced depression-like behaviors and elevation of the hippocampal 5HT_1A_ receptor levels can be recovered by chronic wheel running [115]. Elevated levels of 5-HT_1B_ receptor activity in the hippocampus have been reported in rats subjected to one week of moderate treadmill exercise [112]. Higher levels of 5-HT_1B_ receptor in the hippocampus have been associated with resistance to electric stimulation-elicited learned helplessness [116]. On the contrary, mice with 5-HT_1B_ overexpression in the dorsal raphe nuclei increase anxiolytic behaviors after stress [116]. Both acute and chronic wheel running are known to reduce the level of 5-HT_1B_ receptor mRNA in the dorsal raphe nuclei [113]. These findings support the proposition that exercise is an effective approach to relieve anxiety and protect the brain against uncontrollable stress [93,117,118].

In addition to antidepressant and anxiolytic properties, 5-HT system has also been linked to cognitive function; a perturbation of the 5-HT system is associated with cognitive disease such as Alzheimer’s disease [119,120,121,122]. 5-HT receptors are abundantly distributed throughout cognition-related brain regions, including amygdala, hippocampus and cortex [123,124,125]. The levels of 5-HT_1A_ and 5-HT_2A_ receptors are reduced in brains of mild cognitive impairment patients, indicating that serotonergic dysfunction may represent an early sign of Alzheimer’s disease [119,122,126]. Animals that are subjected to exercise perform better in the acquisition of conditioned learning tasks than their respective controls [111,127]. The improved learning performance induced by exercise is associated with downregulation of 5-HT_1A_ receptor and/or upregulation of 5-HT_2A_ in the hippocampus [111,112,128]. An inhibition of the G_i_ receptor (e.g., 5-HT_1A_) activity and/or a stimulation of the G_s_ receptor (e.g., 5-HT_2A_) activity result in elevation of the levels of cAMP, which is critical for the process of learning and memory. Finally, 5-HT system is known to interplay with the BDNF pathway to form a positive feedback loop that plays an essential role in regulating synaptic plasticity, neurogenesis and neuronal survival in the adult brain [129]. 

## 4. Conclusions

An overwhelming majority of studies accredit that the monoamine systems mediate the exercise-induced enhancement of various brain functions. It is noteworthy that DA, NE and 5-HT receive reciprocal regulation from each other. For instance, 5-HT enhances DA release through the 5-HT_4_ receptors in the striatum [130]. Activation of locus coeruleus induces DA secretion either directly through firing on the ventral tegmental nucleus dopaminergic neurons, or indirectly through the local glutamatergic neuron to activate neurons in the ventral tegmental nucleus [131,132]. Therefore, the cooperative effects of the monoamine family should also be taken into consideration, while studying the effects of exercise on brain function.

The stimulation of the monoamine system is dependent on exercise intensity. Mandatory treadmill exercise and voluntary wheel running are the two most common forms of exercise in rodent models with different exercise intensities. Treadmill exercise is more effective in enhancing the muscle aerobic capacity and in increasing the serum corticosterone level than that of wheel-running exercise [133]. The exercise-associated stress level could be an underlying modulating factor for their differential effects on brain monoamine systems. Different exercise intensities between treadmill exercise and wheel running may induce different degree of feedback in the hypothalamus-pituitary gland-adrenal gland axis. Furthermore, treadmill exercise is much more effective in activating the BDNF signaling pathway than that of wheel running in hippocampus and amygdala [134]. The effects of BDNF cannot be neglected and should be taken into consideration when studying the effects of exercise on monoamine systems.
